# Regional distribution of computed tomography attenuation across the lumbar endplate

**DOI:** 10.1371/journal.pone.0259001

**Published:** 2021-10-27

**Authors:** Kazuyuki Segami, Alejandro A. Espinoza Orías, Hiroe Miyamoto, Koji Kanzaki, Howard S. An, Nozomu Inoue

**Affiliations:** 1 Department of Orthopedic Surgery, Rush University Medical Center, Chicago, Illinois, United States of America; 2 Department of Orthopedic Surgery, Showa University Fujigaoka Hospital, Yokohama, Japan; University of Notre Dame, UNITED STATES

## Abstract

The vertebral endplate forms a structural boundary between intervertebral disc and the trabecular bone of the vertebral body. As a mechanical interface between the stiff bone and resilient disc, the endplate is the weakest portion of the vertebral-disc complex and is predisposed to mechanical failure. However, the literature concerning the bone mineral density (BMD) distribution within the spinal endplate is comparatively sparse. The objective of this study is to investigate the three-dimensional (3D) distribution of computed tomography (CT) attenuation across the lumbosacral endplate measured in Hounsfield Units (HU). A total of 308 endplates from 28 cadaveric fresh-frozen lumbosacral spines were used in this study. Each spine was CT-scanned and the resulting DICOM data was used to obtain HU values of the bone endplate. Each individual endplate surface was subdivided into five clinically-relevant topographic zones. Attenuation was analyzed by spinal levels, sites (superior or inferior endplate) and endplate region. The highest HU values were found at the S1 endplate. Comparisons between the superior and inferior endplates showed the HU values in inferior endplates were significantly higher than those in the superior endplates within the same vertebra and the HU values in endplates cranial to the disc were significantly higher than those in the endplates caudal to the disc within the same disc. Attenuation in the peripheral region was significantly higher than in the central region by 32.5%. Regional comparison within the peripheral region showed the HU values in the posterior region were significantly higher than those in the anterior region and the HU values in the left region were significantly higher than those in the right region. This study provided detailed data on the regional HU distribution across the lumbosacral endplate, which can be useful to understand causes of some endplate lesions, such as fracture, and also to design interbody instrumentation.

## Introduction

The vertebral endplate forms a structural boundary between the intervertebral disc and the trabecular bone in the vertebral body. Comprised of a thin layer of semi-porous subchondral bone, approximately 0.5–1.5 mm thick [1], the principal functions of the endplate are to behave as a physical shield separating the disc from the vertebra [2], to evenly distribute compressive loads to the vertebral body [3], and to constitute the main gateway of nutrient supply from the central endplate to the disc [4, 5]. The dense subchondral bone located at the peripheral region of the endplate, called “ring apophysis” or “epiphyseal ring”, provides secure anchorage for the collagen network of the annulus fibrosus outer layers [6]. As a mechanical interface between the stiff bone and resilient disc, the endplate is the weakest portion of the vertebral-disc complex [7] and is predisposed to mechanical failure [8].

While interbody devices have been increasingly used for lumbar anterior fusion, complications associated with the use of interbody devices have been reported [9–17]. One of the major complications is subsidence of the interbody device into the vertebral body [12–19]. Insufficient strength of the bony endplate has been considered as an important factor for the subsidence; therefore, many investigators have focused on the mechanical strength of lumbar endplate and its relationship with the endplate architecture [1, 9, 20–26].

The mechanical role of the ring apophysis has been acknowledged as support structure for the interbody device. Clinical data supports that the lack of support from the stronger peripheral endplate ring apophysis is a leading cause for artificial disc subsidence [27, 28]. A recent study by Briski *et al*. [29] investigated the mechanical effects of spanning a lateral lumbar interbody cage across the ring apophysis using osteoporotic cadaveric lumbar spines. The authors found that spanning the ring apophysis increased the load to failure by 40% with intact endplate and by 30% with decorticated endplate [29]. These studies demonstrate the importance of endplate strength, especially in the ring apophysis, at the “footprint” of an interbody device.

Because endplate strength cannot be measured directly preoperatively, many investigators have attempted to establish noninvasive methods to estimate the endplate strength using imaging modalities [1, 20–22, 30]. Bone mineral density (BMD) has been considered as a candidate predictor of endplate strength which can be measured by radiographic imaging techniques [21, 30]. Several studies measured trabecular bone BMD in the lumbar vertebral body using dual energy x-ray absorptiometry (DEXA) and/or CT scanning while mechanical testing had been performed on the same specimens [31–33]. Although these studies have shown positive correlations between the endplate strength and the BMD, Hasegawa *et al*. [32] demonstrated that local BMD of subchondral cancellous bone at 5 mm underneath the lumbar endplate determined by a peripheral quantitative CT was better correlated with the mechanical properties as compared with the BMD of the whole lumbar vertebral body measured by DEXA. Noshchenko *et al*. [25] further compared lumbar endplate indentation strength and stiffness with the localized Hounsfield Units (HU) values of the endplate at the same location where the indentation test was performed and showed high correlations between the mechanical properties and the HU values of the lumbar endplate. This study indicates the importance of recording the attenuation values within and underneath the endplate for a better prediction of mechanical properties of the endplate.

Defining the distribution of HU values within the three-dimensionally curved thin endplate is technically demanding using currently available clinical imaging modalities, even though various reformatting procedures are available these days. As a method to measure HU distribution of the subchondral bone under the curved joint surface, Müller-Gerbl and colleagues first demonstrated the use of CT osteoabsorptiometry [34]. Using this method, the HU distribution of the cervical endplate itself was first successfully measured using CT osteoabsorptiometry based on clinical CT images, inferring BMD patterns in the cervical endplate [35]. However, to the best of our knowledge, HU distribution within the lumbar endplate across the endplate surface has not been investigated in the literature. Therefore, the purpose of the present study was to investigate the HU distribution obtained from clinical CT across the lumbar endplate. In order to compare published endplate structural properties such as endplate thickness and indentation strength, the HU distribution was analyzed by spinal level, site (superior or inferior endplate) and relevant anatomical regions within an endplate.

## Materials and methods

### Specimens

A total of 308 endplates of 28 fresh-frozen lumbar spines (L1-S1) from deceased donors (19 females and 9 males; mean age 62.7 years old; range of ages, 31–76 years old) were used in this study. Each lumbar spine was screened by plain X-rays first to exclude the specimen with deformity, tumors, severe osteoporosis and endplate compression fracture. After the screening, the specimens were wrapped in moist towels and placed into a labeled, plastic bags before being stored frozen at -20°C. This study was exempted by the IRB at Rush University Medical Center since it only included cadaveric specimens.

### Creation of endplate surface model

Each spine was CT-scanned (BrightSpeed, GE Healthcare, Waukesha, WI, tube voltage: 120 kV, tube current: 250 mAs, field of view: approximately 200 mm, image matrix: 512×512, slice thickness: 0.625 mm, no spacing). Raw imaging data of axial slices were exported in the DICOM format. The CT images were imported into a 3D reconstruction software package (Mimics 22 Research, Materialise Inc., Leuven, Belgium), and 3D surface mesh models from L1 to S1 were created using a preset bone threshold level of 250 HU. Both superior and inferior endplate mesh models were further segmented from each vertebral 3D model excluding remarkable osteophytes and converted to a point-cloud model using custom-written Visual C++ with MFC environment software.

### Determination of attenuation in HU across the endplate

**Fig 1:**
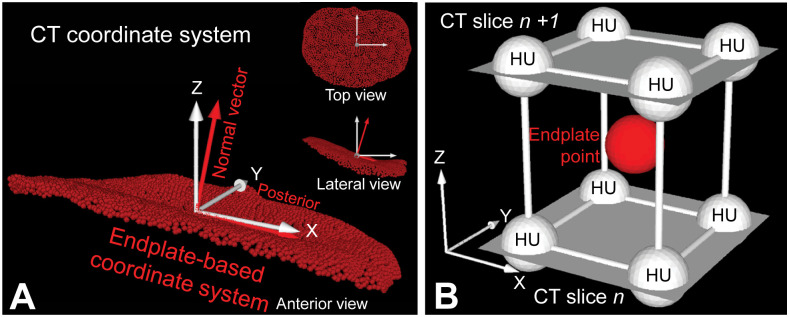
Endplate point-cloud surface model and determination of attenuation in Hounsfield Units (HU) at individual point of the model. A: point-cloud model (*red points*), endplate-based coordinate system (*red arrows*) and CT coordinate system (*white arrows*). B: 3D spatial relationship between an arbitrary point of the endplate point-cloud model (*red point*) and 8 adjacent points in the 2 adjacent CT images (white points). White arrows; CT coordinate system.

**Fig 2:**
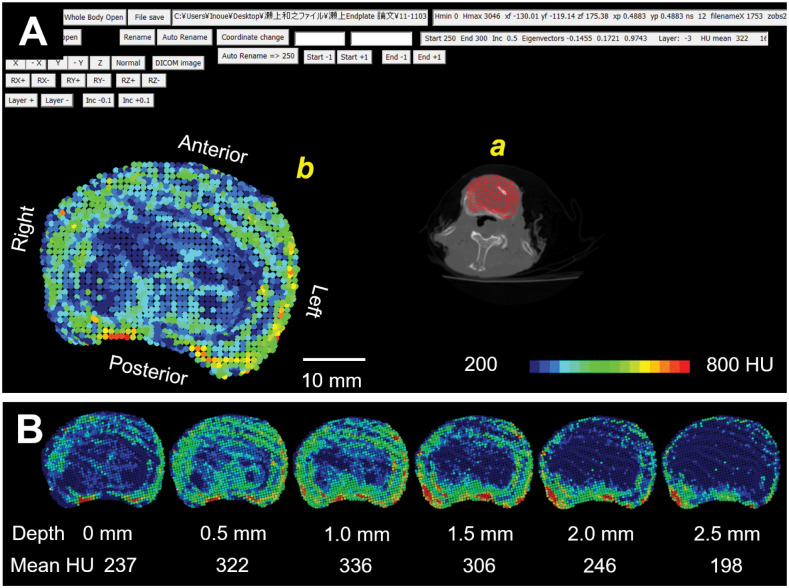
Measurement of Hounsfield Units (HU) across the endplate. A: screenshot of custom-written software for measurement to determine HU values at individual points consisting of a 3D endplate point-cloud model determined by a DICOM dataset. *a;* an axial CT slice (note; only one slice is shown on the screen although multiple slices are involved in the measurement). Red dots indicate location of the endplate point-cloud model. *b;* HU distribution on the endplate point-cloud model. Position and orientation of the point-cloud model can be changed in any increment. B: An example of changes in HU distribution and mean HU values at the surface and at depths 0.5–2.5 mm underneath the endplate surface.

In order to obtain the attenuation distribution measured in HU within and underneath the endplate, the endplate point-cloud surface was virtually moved perpendicular to the endplate surface towards the vertebral body in increments of 0.5 mm up to 2.0 mm. This was done considering partial volume effects and the lumbar endplate thickness of approximately 0.5–1.5 mm as described in the literature [1] to obtain a HU value at each depth. To determine the moving direction of the endplate, the normal vector of the endplate surface was calculated based on the individual eigenvectors of the endplate surface mesh model (Fig 1A). Each point of the endplate point-cloud model was moved in a direction opposite to the normal vector. At each point-cloud point, the corresponding HU value was calculated by trilinear interpolation of HU values at 8 adjacent volumes in 2 axial CT slices adjacent to each point (Fig 1B) [36, 37]. A mean value of the HUs of the all points in the endplate point-cloud model was calculated at each depth (Fig 2). The mean HU value within its range (0.5–2.0 mm) was defined as the HU value for the endplate.

### Determination of endplate topographic zones

**Fig 3:**
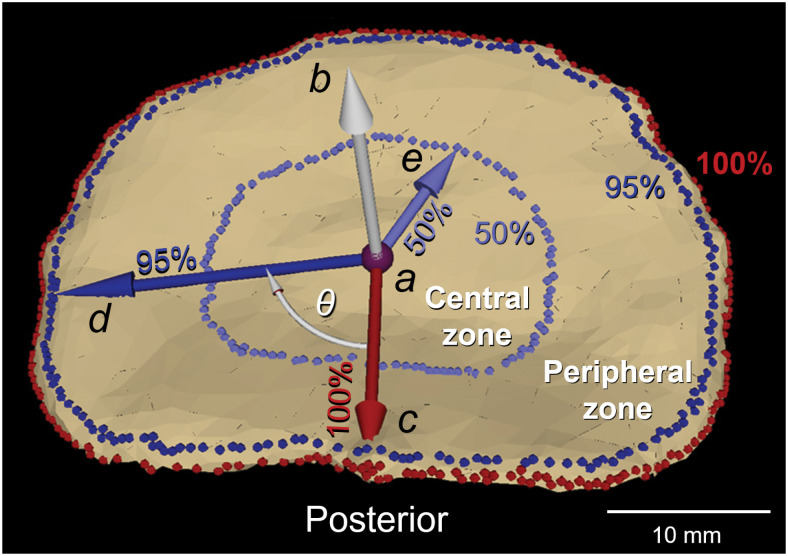
Determination of central and peripheral zones based on endplate-based spherical coordinate system. *a*: centroid of the endplate and origin of the endplate-based polar coordinate system used to define the endplate zones (0° coincides with posterior direction and positive angle values are clockwise). *b*: endplate normal vector. *c*: vector pointing towards the most posterior point of the endplate. (note: penetrating arrowhead demonstrates the 3D nature of the endplate surface) *d*: a vector with a length of 95% of the maximum radius. *e*: a vector with a length of 50% of the maximum radius. *θ*: angular parameter. *Red dots*: outermost margin. *Dark blue dots*: concentric margin contracted to 95% of the outer margin. *Light blue dots;* concentric margin contracted to 50% of the outer margin.

**Fig 4:**
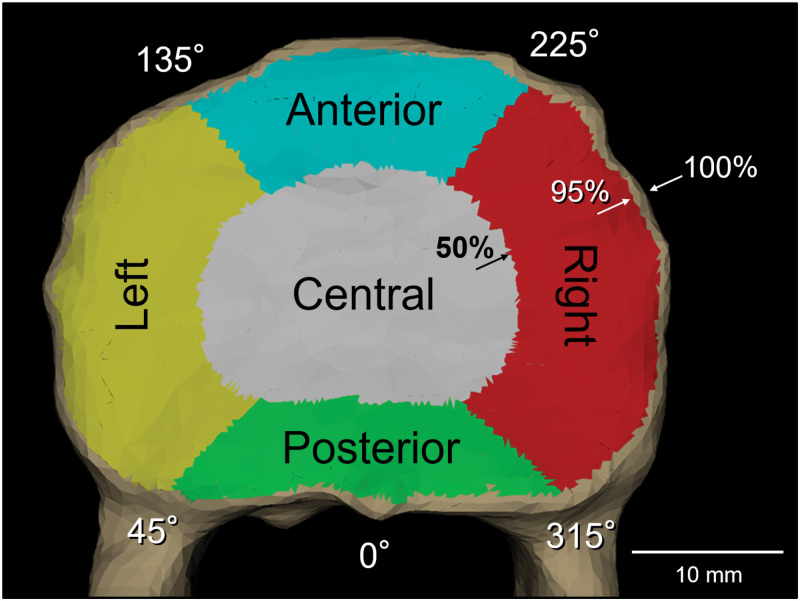
Definition of the five topographic zones, superimposed on a vertebra surface model. Note that osteophytes mesh elements with over 45° tilt angle and the most outer margin of the endplate where the cortical wall of the vertebral body exists underneath the endplate are not included in the peripheral zones.

An endplate-based local coordinate system was used to establish a zone system on the endplate (Figs 1A and 3). The centroid and a normal vector of the entire endplate surface were calculated [31]. Mesh elements at the edge of the endplate were automatically excluded when the angle between their normal vector and the endplate normal vector was over 45°, in order to exclude osteophytes. The position of each mesh element, initially described in Cartesian coordinates was translated into spherical coordinates with the centroid of the endplate as its origin. The mesh elements at the edge of the endplate were detected (red dots in Fig 3) and radius of each mesh element was normalized by the largest radius for each angle. Mesh elements with over 95% of the largest radius were not included for analysis in order to exclude osteophytes and the cortical wall of the vertebral body underneath the endplate edge. Based on the radius of the quasi-elliptical footprint of the endplate, two concentric zones were defined: i) a radial range between 95% and 50% of the radius outlined the peripheral zone, and ii) when the radius was less than 50%, it provided a contour containing the central zone (Figs 3 and 4). The peripheral zone was further divided into four anatomically-relevant zones: posterior, left, anterior and right zones, based on angles from a base axis oriented towards the most posterior point of the endplate (Fig 4) [38, 39]. The actual distances for the 50% and 90% radii for each zone are shown in Table 1.

**Table 1:** Actual distance for 50% and 95% radii by anatomical zone.

Level	Site	Post 50%		Left 50%		Ant 50%		Right 50%		Post 95%		Left 95%		Ant 95%		Right 95%	
		Radius	SD	Radius	SD	Radius	SD	Radius	SD	Radius	SD	Radius	SD	Radius	SD	Radius	SD
L1	Sup	9.29	1.00	11.00	1.01	9.35	0.77	11.12	0.86	17.64	1.92	20.91	1.90	17.76	1.46	17.76	1.46
	Inf	9.60	1.08	11.53	1.02	9.76	1.05	11.37	0.98	18.22	2.03	21.90	1.90	18.55	2.01	18.55	2.01
L2	Sup	9.76	1.36	11.43	1.02	9.81	0.85	11.76	0.99	18.53	2.57	21.72	1.94	18.65	1.62	18.65	1.62
	Inf	9.82	1.01	12.00	1.03	10.18	0.99	12.03	1.13	18.67	1.92	22.81	1.94	19.34	1.88	19.34	1.88
L3	Sup	10.20	1.37	12.03	1.15	10.07	0.87	12.21	0.99	19.38	2.61	22.86	2.20	19.17	1.67	19.17	1.67
	Inf	9.76	0.99	12.38	1.03	10.14	1.17	12.48	1.23	18.57	1.89	23.50	1.98	19.27	2.23	19.27	2.23
L4	Sup	10.40	1.26	12.50	1.29	10.08	0.91	12.56	1.07	19.75	2.40	23.76	2.45	19.16	1.74	19.16	1.74
	Inf	9.72	0.97	12.66	1.26	10.06	1.22	12.74	1.51	18.48	1.86	24.06	2.38	19.13	2.30	19.13	2.30
L5	Sup	10.51	1.43	13.10	1.26	9.93	1.18	13.04	1.51	19.97	2.74	24.89	2.39	18.87	2.24	18.87	2.24
	Inf	9.57	1.07	12.88	1.36	9.89	1.27	13.05	1.42	18.19	2.03	24.48	2.59	18.79	2.43	18.79	2.43
S1	Sup	10.15	1.44	12.93	1.33	8.56	1.11	13.09	1.45	19.27	2.73	24.56	2.52	16.28	2.12	16.28	2.12

*Note*: *Post 50%*: Distance for 50% radius for posterior zone, *Left 50%*: Distance for 50% radius for left zone, *Ant 50%*: Distance for 50% radius for anterior zone, *Right 50%*: Distance for 50% radius for right zone, *Post 95%*: Distance for 95% radius for posterior zone, *Left 95%*: Distance for 95% radius for left zone, *Ant 95%*: Distance for 95% radius for anterior zone, *Right 95%*: Distance for 95% radius for right zone, *Sup*: superior endplate, *Inf*: inferior endplate,

### Statistical analyses

ANOVA with Tukey’s *post hoc* test was used to evaluate differences between zones and spinal levels. Differences between superior and inferior endplates in the same vertebrae or same intervertebral disc were carried out with a paired Student’s *t*-test. Results were presented as mean ± SD. Significance was set at p < 0.05.

## Results

### Endplate attenuation compared by gender

A comparison of the HU values of both superior and inferior endplates between females and males showed no significant differences from L1 to S1 (Table 2).

**Table 2:** Comparison by gender.

	Superior endplate				Inferior endplate			
	Female	Male	Mean	SD	Female	Male	Mean	SD
**L1**	303.6	302.7	303.3	98.8	333.4	364.3	343.3	84.4
**L2**	288.7	306.1	294.3	114.7	345.9	399.7	363.2	100.4
**L3**	287.8	301.2	292.1	109.0	372.5	411.5	385.0	111.0
**L4**	301.8	313.2	305.5	110.4	404.1	409.2	405.7	106.0
**L5**	342.3	317.6	334.3	110.9	402.3	390.2	398.4	88.7
**S1**	429.3	384.0	414.8	115.2				

### Attenuation values across different spinal levels

**Fig 5:**
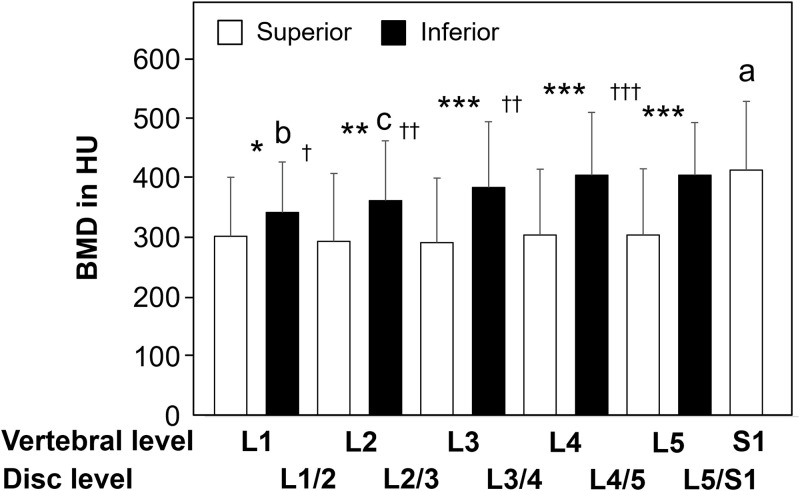
Comparison of the endplate BMD among different spinal levels and between the superior and inferior endplates within the same vertebra or same intervertebral disc. a: p<0.0001 compared with superior endplates at L1, L2, L3 and L4. p<0.0005 compared with superior endplates at L5. compared with inferior endplate at L1 (p<0.0007) and L2 (p<0.05). b: compared with inferior endplates at L3 (p<0.009), L4 (p<0.005) and L5 (p<0.003). c: p<0.03 compared with inferior endplate at L3. *: p<0.008 between superior vs. inferior endplates in the same vertebra. **: p<0.0005 between superior vs. inferior endplates in the same vertebra. ***: p<0.0001 between superior vs. inferior endplates in the same vertebra. †: p<0.0004 between superior vs. inferior endplates in the same disc. ††: p<0.0001 between superior vs. inferior endplates in the same disc. †††: p<0.0002 between superior vs. inferior endplates in the same disc.

HU values of superior endplates at the different spinal levels showed no significant differences from L1 to L5. However, attenuation at the superior endplate at S1 was significantly higher than the HU values at L1 through L5 (p < 0.0001 at L1, L2, L3 and L4 superior endplate; p < 0.0005 at L5 superior endplate) (Fig 5). The HU values of the inferior endplate at L1 were significantly lower than those at L3 (p < 0.009), L4 (p < 0.005) and L5 (p < 0.003). The HU values of the inferior endplate at L2 were significantly lower than those at L3 (p < 0.03) (Fig 5).

### Attenuation compared between the superior and inferior endplates within the same vertebra or same intervertebral disc

Within the same vertebra, the HU values of inferior endplates were significantly higher than those of superior endplates in all lumbar spinal levels (p < 0.008 at L1; p < 0.0005 at L2; p < 0.0001 at L3, L4 and L5) (Fig 5). Within the same intervertebral disc, the HU values of endplates cranial to disc were also significantly higher than those of endplates caudal to disc in all spinal levels (p < 0.0004 at L1/2; p < 0.0001 at L2/3 and L3/4; p < 0.0002 at L4/5) except L5/S1 (p = 0.24) (Fig 5).

### Attenuation within an endplate

**Fig 6:**
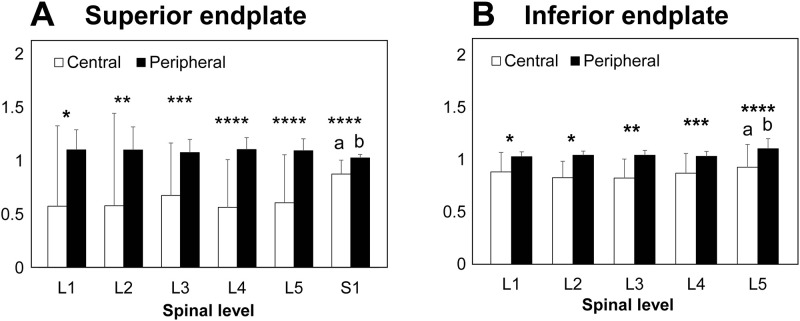
A. Comparison of the BMD in the central and peripheral zones of the superior endplate among different spinal levels and within the superior endplate. a: p<0.009 compared with central region of the superior endplates at L4. p<0.05 compared with central region of the superior endplates at L5. b: p<0.009 compared with peripheral region of the superior endplates at L4. p<0.05 compared with peripheral region of the superior endplates at L5. *: p<0.007 between central vs. peripheral regions in the same spinal level. **: p<0.02 between central vs. peripheral regions in the same spinal level. ***: p<0.002 between central vs. peripheral regions in the same spinal level. ****: p<0.0001 between central vs. peripheral regions in the same spinal level. B. Comparison of the BMD in the central and peripheral zones of the inferior endplate among different spinal levels and within the inferior endplate. a: p<0.03 compared with central region at L3. b: compared with peripheral region at L1 (p<0.02), L2 (p<0.05) and L4 (p<0.003). *: p<0.003 between central vs. peripheral regions in the same spinal level. **: p<0.0001 between central vs. peripheral regions in the same spinal level. ***: p<0.002 between central vs. peripheral regions in the same spinal level. ****: p<0.0007 between central vs. peripheral regions in the same spinal level.

**Fig 7:**
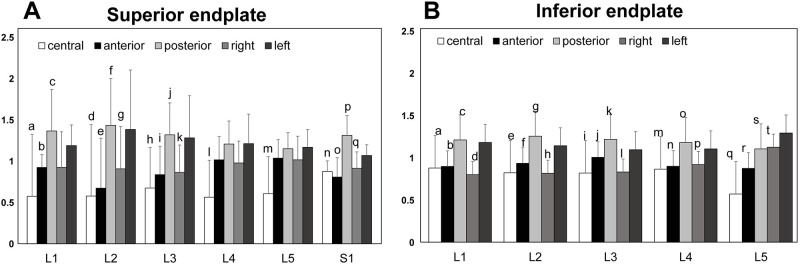
A. Comparison of the regional BMD in the superior endplate. Central: central zone, anterior: anterior zone, posterior: posterior zone, right: right zone, left: left zone. a: compared with Pos (p<0.02) and Left (p<0.009) at L1. b: compared with Pos (p<0.002) and Left (p<0.004) at L1. c: compared with Right (p<0.01) at L1. d: compared with Pos (p<0.03) at L2. e: compared with Pos (p<0.02) at L2. f: compared with Right (p<0.005) at L2. g: compared with Left (p<0.04) at L2. h: compared with Pos (p<0.002) and Left (p<0.02) at L3. i: compared with Pos (p<0.006) at L3. j: compared with Right (p<0.005) at L3. k: compared with Left (p<0.04) at L3. l: compared with Ant (p<0.006), Pos (p<0.0003), Right (p<0.008) and Left (p<0.0001) at L4. m: compared with Ant (p<0.006), Pos (p<0.0004), Right (p<0.02) and Left (p<0.0003) at L5. n: compared with Pos (p<0.0001) and Left (p<0.0004) at S1. o: compared with Pos (p<0.0001) and Left (p<0.003) at S1. p: compared with Right (p<0.0002) and Left (p<0.0004) at S1.q: compared with Left (p<0.008) at S1. B. Comparison of the regional BMD in the inferior endplate. Central: central zone, anterior: anterior zone, posterior: posterior zone, right: right zone, left: left zone. a: p<0.0001 compared with Pos and Left at L1. b: compared with Pos (p<0.002) and Left (p<0.0009) at L1. c: compared with Right (p<0.0001) at L1. d: compared with Left (p<0.0004) at L1. e: p<0.0001 compared with Pos and Left at L2. f: compared with Pos (p<0.0008), Right(p<0.04) and Left (p<0.006) at L2. g: compared with Right (p<0.0003) at L2. h: compared with Left (p<0.002) at L2. i: compared with Ant (p<0.009), Pos (p<0.0001) and Left (p<0.005) at L3. j: compared with Pos (p<0.007) and Right(p<0.02) at L3. k: compared with Right (p<0.0001) at L3. l: compared with Left (p<0.02) at L3. m: compared with Pos (p<0.0001) and Left (p<0.0004) at L4. n: compared with Pos (p<0.0001) and Left (p<0.001) at L4. o: compared with Right (p<0.0004) at L4. p: compared with Left (p = 0.0644) at L4. q: compared with Ant (p<0.009), Pos (p<0.002), Right(p<0.0001) and Left (p<0.0001) at L5. r: compared with Pos (p<0.05), Right(p<0.0001) and Left (p<0.0001) at L5. s: compared with Left (p<0.006) at L5. t: compared with Left (p<0.04) at L5.

Overall, the HU values in the peripheral zones (including anterior, right, posterior and left) were significantly higher than those in the central zone by 32.5% in average (Fig 6A and 6B). Analyzing by peripheral zones, the HU values in the posterior zone were significantly higher than those of anterior zone at all levels (p < 0.002 at L1 superior and inferior endplate, p < 0.02 at L2 superior endplate, p < 0.006 at L3 superior endplate, p < 0.0001 at S1 superior endplate and L4 inferior endplate, p < 0.0008 at L2 inferior endplate, p < 0.007 at L3 inferior endplate, p < 0.05 at L5 inferior endplate) except L4 and L5 superior endplate (p = 0.16 and 0.26, respectively) (Fig 7A and 7B). The HU values of the left zone were significantly higher than those of the right zone at all levels (p < 0.04 at L2, L3 superior endplate and L5 inferior endplate; p < 0.008 at S1 superior endplate; p < 0.0004 at L1 inferior endplate; p < 0.002 at L2 inferior endplate; p < 0.02 at L3 inferior endplate; P = 0.0644 at L4 inferior endplate) except L1, L4 and L5 superior endplate (p = 0.16, 0.23 and 0.28, respectively) (Fig 7A and 7B). The HU values from the anterior to the right zone were lower, whereas HU values were higher from the posterior to the left zone (Fig 7A and 7B).

## Discussion

The present study demonstrated considerable regional variation within each endplate with lower attenuation recorded in the central zone and higher HU values found in the peripheral regions. Similar findings have been reported by other authors regarding thickness distribution of the lumbar endplates, showing the thickness of the endplate was greater at the margins than in the central regions (Table 3) [1, 20–22]. Previous biomechanical studies on the lumbar endplate also showed indentation strength and stiffness were higher at peripheral, especially posterolateral, and lower in the center of the lumbar endplates (Table 3) [9, 23, 24]. The higher HU values in the peripheral region may be explained by existence of a ring apophysis in this region.

**Table 3:** Studies on lumbar endplate thickness, strength and bone mineral density (BMD).

Authors	Spinal level	Method	Level effect	Peri vs. Cent	Ant vs. Post	Sup vs. Inf ^a^	Cranial to disc vs. caudal to disc ^b^
*Thickness*							
Roberts [20]	L1-S1	Radiograph	NA	Peri > Cent	NA	NA	Cranial > Caudal
Zhao [21]	T8-L5	Micro-radiograph	Lower > Upper ^c^	Peri > Cent	NA	Inf > Sup	NA
Edwards [1]	T1, T5, L1, L5	Macroscopic	Lower > Upper	Peri > Cent	NA	NA	NA
Hulme [22]	T9-L5	Micro-CT	NA	Peri > Cent	Post > Ant	NA	Cranial > Caudal ^d^
Wang [26]	L1-L5	Micro-CT	Lower > Upper ^c^	NA	NA	NA	Cranial > Caudal
*Strength*							
Grant [23]	L3-S1	Indentation	S1 > Lumbar sup	PL > Cent	Post > Ant	Inf > Sup	NA
Lowe [9]	T1-L5	Indentation	NA	PL> Cent	NA	NA	NA
Oxland [24]	L3-L5	Indentation	NA	PL> Cent	Post > Ant	NA	NA
Noshchenko [25]	L1-L5	Indentation	Lower > Upper	NA	Post > Ant	Inf > Sup	NA
*BMD*							
Lu [30]	L1-S1	QCT ^e^	S1 > L3,4,5 > L1,2	NA	NA	NA	NA
Zhao [21]	T8-L5	Micro-radiograph ^f^	NA	Peri > Cent	NA	NA	NA
Noshchenko [25]	L1-L5	Micro-CT, CT ^g^	NA	NA	NA	NA	NA
Wang [26]	L1-L5	Micro-CT	NA	NA	NA	NA	Cranial > Caudal

***NOTES*** *Peri*: peripheral zones, *Cent*: central zone, *Ant*: anterior zone, *Post*: posterior zone, *Sup*: superior endplate, *Inf*: inferior endplate, *NA*: data not available, *Cranial*: endplate cranial to disc, *Caudal*: endplate caudal to disc, *Lower*: lower lumbar levels, *Upper*: upper lumbar levels, *PL*: posterolateral zones.

^a^ within the same vertebra.

^b^ within the same vertebral disc.

^c^ only inferior endplate.

^d^ except the anterior ring apophysis.

^e^ BMD in transverse layers adjacent to the endplate.

^f^ optical density, surrogate for BMD.

^g^ HU was translated into density units (mg/mm³).

Our results show that attenuation in the posterior region was always higher than in the anterior region in all levels. Hulme et al. [22], evaluated regional variation in vertebral bone morphology using micro-CT and found that posterior regions of the vertebrae had greater bone volume, more connections, reduced trabecular separation and more plate-like isotropic structures than their corresponding anterior regions. Trabecular tracts running obliquely from the superior process downward to the inferior endplate and from the inferior process upward to the superior endplate through the pedicle were illustrated in the literature as early in 1925 by Gallois and Japoit [40]. Dense trabecular tracts extending from the pedicles were also shown by soft x-ray images [41]. The trabecular bones from the pedicles may contribute to increase attenuation in the posterior endplate.

Unexpectedly, significant differences were found between the right and left peripheral regions with higher HU values in left side than those in the right side by about 24%. Asymmetry of the vertebral shape has been reported by Masharawi *et al*. [42] They investigated the vertebral body shape of 240 normal adult thoracolumbar spines in the Hamann-Todd Human Osteological Collection and reported that 92% of all women and 86% of all men had vertebral bodies which showed greater lateral height in the right side than in the left, in a phenomenon termed by the authors *wedging* towards the left. However, this research team also measured dimensions of the epiphyseal ring of the same specimens and reported no significant differences between the right ring diameter normalized by vertebral body width and that in the left ring [6]. Since attenuation distribution in joints and the endplate has been thought to represent loading history, the higher HU values in the left side of the lumbar endplate may indicate that higher load is applied to the left side of the endplate with the same epiphyseal ring area. Since the present study used cadaveric lumbar spines; therefore, future *in vivo* studies will be needed to investigate relationships between 3D lumbar curvature and asymmetrical HU distribution to prove this hypothesis.

In the comparison of the attenuation between the superior and inferior endplates within the same vertebral body, the HU values of inferior endplates were always significantly higher than those of superior endplates. When the endplate attenuation was compared within the same intervertebral disc, the HU values in the endplate cranial to the disc were higher than those in the endplate caudal to the disc except in the L5/S1 disc level. These results agree with the previous studies on other structural properties of the lumbar endplate such as thickness and strength of the endplate (Table 3). These findings may explain higher incidence of compression and burst fractures and interbody subsidence in the superior endplate (endplate caudal to the disc) as compared with the inferior endplate (endplate cranial to the disc) [12, 43].

In the present study, while the HU values of the superior endplate did not show statistically significant differences from L1 to L5, a significant increase was found from L5 to S1. Gradual increase in loading is expected with each successive lower spinal level; in fact, several studies showed vertebral compression strength gradually increases from C3 to L5 [44, 45]. Similarly, endplate surface area has also been reported to increase with more caudal spinal levels [39, 41]. Since attenuation reflects bone mineral density (*i*.*e*., bone mineral content per unit volume), an increase in endplate area provides an increase in total mineral content per endplate, which may constitute functional adaptation to the increased load in the lower lumbar levels. In comparable results to our study, Weaver and Chalmers showed approximately the same BMD determined by ash weight in L3, L4 and L5 vertebral trabecular bones [46]. They speculated that vertebral strength variations with the spinal level were attributable to vertebral size, rather than BMD changes. These two theses may be further supported by significant increases in BMD and decreases in endplate area at the sacrum endplate [39], in which the increased BMD may compensate for the reduced endplate surface area in S1. Further experimental studies comparing stress (load per unit area) and BMD distribution in the endplate will be required to prove this theory.

The main limitation of the present study was the use of a small number of rather old age (average age: 62.7) and mostly female cadaveric spines. Some degrees of spinal degeneration present in our spines and as such our dataset may not represent healthy lumbar spine BMD. Other inherent limitations stemming from the use of cadaveric specimens, are the absence of data on low back pain symptoms and spinal alignment.

The zone system in the present study set a border line between the central and peripheral regions at 50% of the radius (25% of outer dimensions). The width of the epiphyseal ring was reported as less than 20% of the outer dimensions of the endplate [6]; therefore, the peripheral zone in our zone system should include the epiphyseal ring. However, if the peripheral region was set more peripherally, the HU values in the peripheral regions would be higher than the results presented here. Since our zoning system uses the spherical coordinate system, any radius and angular parameters can be selected for determination of the zones. Furthermore, the border of the epiphyseal ring could be determined by referring the HU distribution if an appropriate threshold value is identified. Future studies will investigate relationships between HU distribution and surface 3D geometry of the endplate to identify the epiphyseal ring area in the HU distribution map.

The present study used the 3D endplate model segmented from the 3D CT vertebral models as a template of the endplate surface to obtain endplate surface HU values. Considering clinical application of our technique to obtain the HU values at the footprint of the interbody device, the endplate model can be replaced by a 3D model of the device as shown by Chahla *et al*. [36] who used 3D device models to obtain HU values on the surface and adjacent area of the device for postoperative evaluation of device fixation. Current progress on preoperative planning and simulation techniques would allow virtual placement of the device directly into the CT 3D space without the 3D vertebra surface model. Given a 3D model of the interbody device is provided by a manufacturer and position and orientation of the device are determined in the DICOM dataset, HU distribution of the device surface and any location around the device surface could be measured preoperatively and postoperatively using a conventional clinical CT machine by using the technique described in the present study.

## Conclusions

Three-dimensional distribution of CT attenuation expressed in Hounsfield Units (HU) across the lumbar endplate measured by clinical CT revealed the most significant regional difference between the peripheral and central regions of the endplate with 32.5% higher HU values in the peripheral region, demonstrating the importance of the peripheral region in mechanical support for the interbody device.

## Supporting information

S1 Data (XLSX)
